# Successful transurethral resection of the prostate in ectopic prostate pheochromocytoma: A case report

**DOI:** 10.1097/MD.0000000000019852

**Published:** 2020-04-24

**Authors:** Jin Zhou, Wen-Feng Wu, Wenbin Zhang, Jun Xin, Wen-Hui Lei

**Affiliations:** a Department of Medicine, Lishui Municipal Central Hospital, Lishui, Zhejiang Province; b Department of Urology, The first hospital of Quanzhou affiliated to Fujian Medical University, Quanzhou, Fujian, China.

**Keywords:** ectopic prostate pheochromocytoma, extra-adrenal pheochromocytoma, pheochromocytoma

## Abstract

**Introduction:**

Most pheochromocytomas of the urinary tract are located in the bladder. However, ectopic prostate pheochromocytomas have rarely been reported. We herein report an unusual case of ectopic prostate pheochromocytoma successfully treated by transurethral resection of the prostate (TURP).

**Patient concerns:**

A 44-year-old Asian man with no significant previous medical history such as hypertension, presented to the urologist complaining of palpitations and anxiety on urination for more than 1 month.

**Diagnoses:**

Pathological examination confirmed ectopic prostate pheochromocytoma.

**Interventions:**

An ectopic prostate pheochromocytoma without definite metastasis was confirmed. The lesion was successfully treated via TURP.

**Outcomes:**

All of his symptoms completely and immediately disappeared after surgery. Over a 21-month follow-up period, a repeat abdominal computed tomography (CT) scan did not show any evidence of recurrence.

**Conclusion:**

When patients present with symptoms of catecholamine excess on urination, extra-adrenal pheochromocytoma in the prostate should also be considered. TURP may be a viable option for therapy.

## 1 Introduction

Pheochromocytomas are neural crest-derived tumors that produce, metabolize, and secrete catecholamines. The clinical symptoms of pheochromocytomas can vary from common signs such as fatigue and headache to unusual symptoms such as paroxysmal hypertension, palpitations, and abdominal or chest pain.^[1]^ The varied symptoms of pheochromocytomas reflect the metabolic actions of the catecholamines.^[2]^

According to the 2004 World Health Organization classification, pheochromocytoma can be divided into intra- and extra-adrenal chromaffin cell tumors, with 80% to 85% arising from adrenal medullary chromaffin tissue and 15% to 20% occurring in extra-adrenal chromaffin tissues.^[3]^ Extra-adrenal pheochromocytomas in the abdomen are usually located at the aortic bifurcation or the inferior mesenteric artery where chromaffin tissues are collected.^[4]^ However, to our knowledge, ectopic prostate pheochromocytomas have rarely been reported. We herein report a rare case of ectopic prostate pheochromocytoma presenting with palpitations on urination and successfully treated by transurethral resection of the prostate (TURP).

## 2 Case presentation

**Figure 1:**
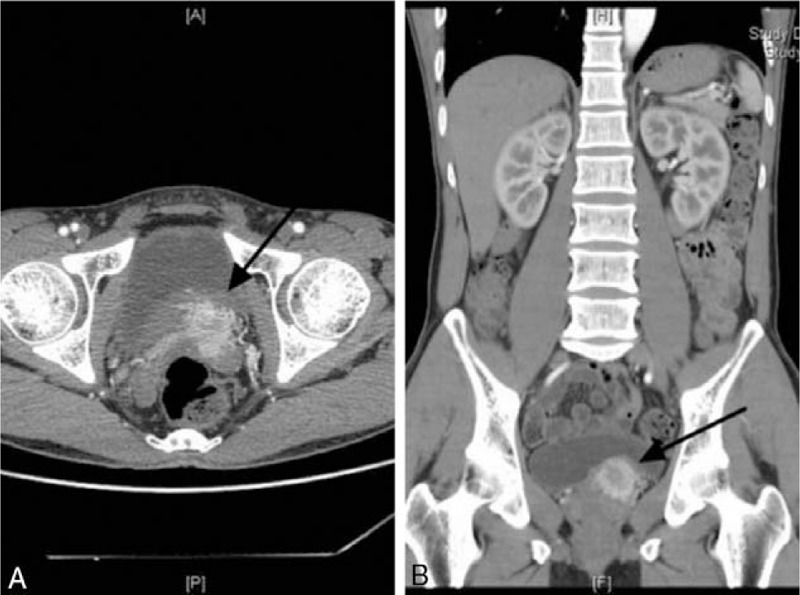
Contrast enhanced computed tomography (CT): Transverse (A) and frontal (B) views reveal a 29 mm nodular lesion on the left prostate (arrows).

**Figure 2:**
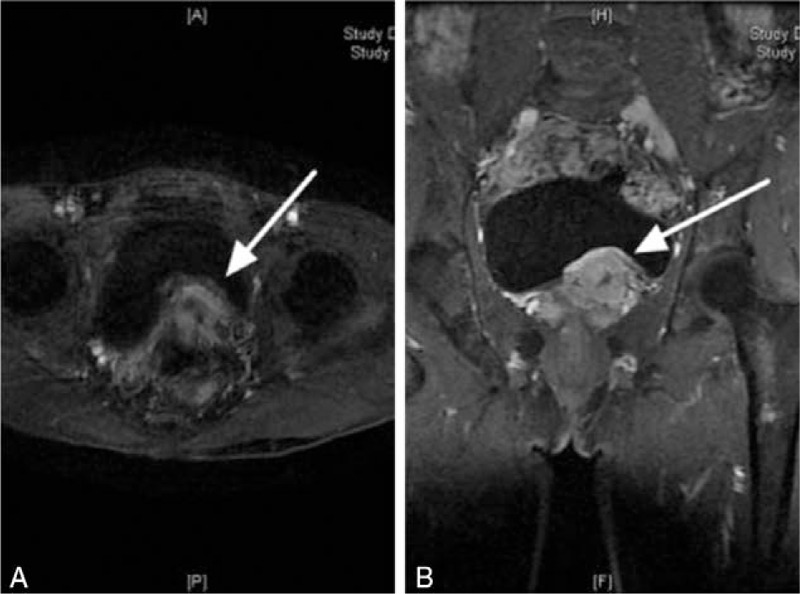
Magnetic resonance imaging (MRI): Transverse (A) and frontal (B) views indicate prostate tumor involving local central lobe (arrows).

A 44-year-old Asian man presented with palpitations and anxiety on urination for more than 1 month. He had no significant previous medical history such as hypertension. On examination, temperature was 36.5°C, heart rate was 112 beats per minute, respirations were 20 breaths per minute, and blood pressure was 126/78 mm Hg. Digital rectal examination revealed a mildly tender mass in the left lobe of the prostate. No obvious abnormalities were found in cardiopulmonary and abdominal examinations. Laboratory tests revealed serum metanephrine 575 pg/ml (normal range: 15–475 pg/ml), serum normetanephrine 160 pg/ml (normal range: 30–95 pg/ml), and 24-hour urine catecholamine 152 μg/24 hour (normal range: 13–42 pg/ml). The contrast enhanced computed tomography (CT) scan revealed a 51 × 38 mm^2^ slightly irregular prostate. We also found a 29 mm nodular lesion of marked enhancement in the arterial stage on the left prostate (Fig. 1). A magnetic resonance imaging (MRI) -enhanced prostate scan was recommended to exclude the possibility of prostate malignancy. MRI indicated the possibility of a malignant prostate tumor involving the local central lobe (Fig. 2).

**Figure 3:**
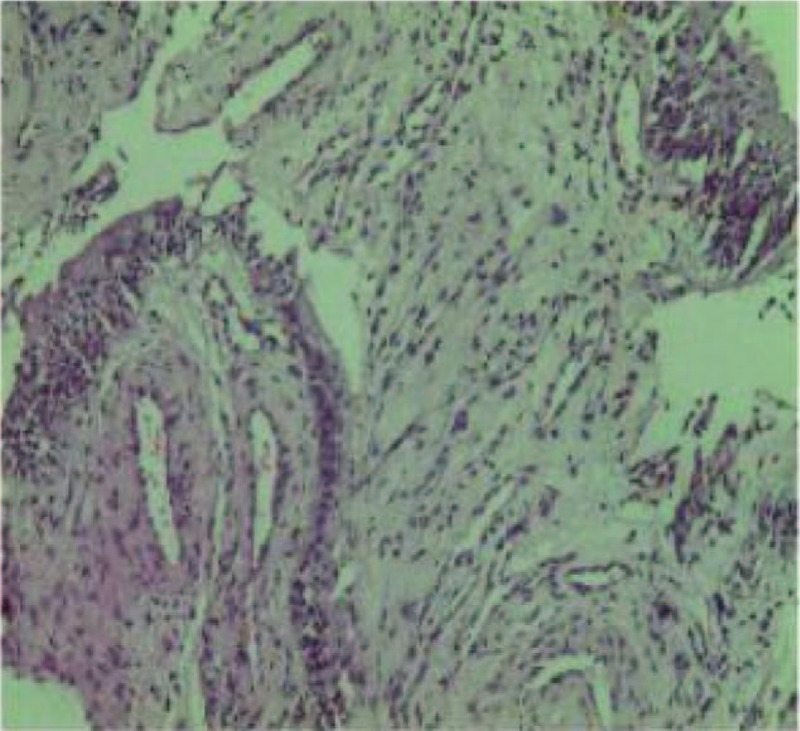
Bladder biopsy: Biopsy shows chronic inflammation of the mucosa, interstitial vascular hyperplasia, and dilatation (HE staining, ×40).

**Figure 4:**
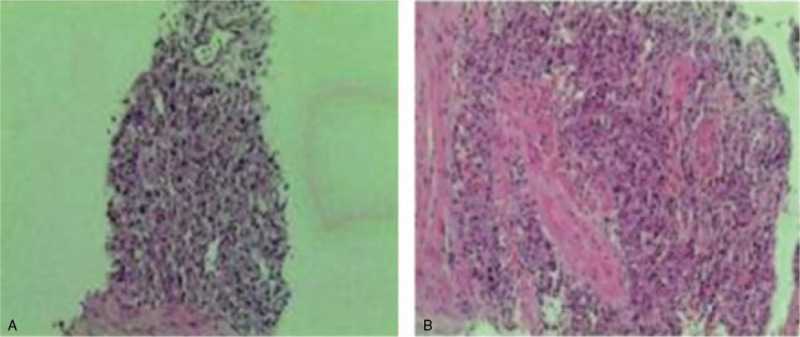
Prostate biopsy (HE staining): A (×40), B (×100), biopsy suggests adrenal extrarenal pheochromocytoma.

Cystoscopy revealed a broad-based mass at the left bladder neck, about 2 × 2 cm^2^ in size, with follicular changes on the surface of the mass, protruding into the bladder cavity, and easily hemorrhaged on the surface. Bladder biopsy showed chronic inflammation of the mucosa, interstitial vascular hyperplasia and dilatation (Fig. 3). Cystoscopy could not determine whether the mass was a bladder or prostate tumor, so we decided to do a transrectal prostate biopsy. Pathological examination of the prostate biopsy suggested adrenal extrarenal pheochromocytoma (Fig. 4). All results suggested that this pheochromocytoma was a benign tumor.

**Figure 5:**
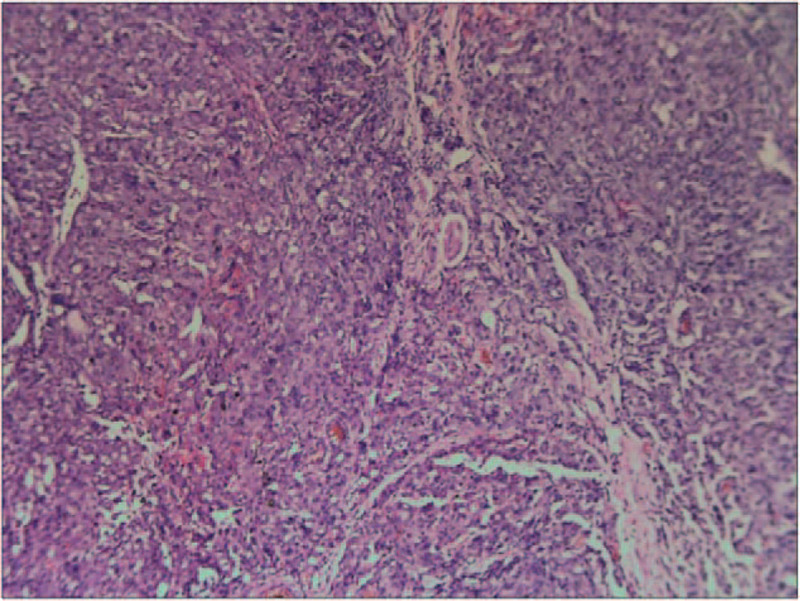
Pathological examination (HE staining (×100)) of prostate: Examination confirms adrenal extrarenal pheochromocytoma.

Treatment methods were discussed with oncologists. Laparoscopic surgery or transurethral resection of the tumor was recommended for the patient. The patient refused resection via laparoscopic surgery, so transurethral resection of the tumor was performed. Before surgery, the patient was administered 10 mg oral phenoxybenzamine. During the operation, a solid mass was found in the left lobe of the prostate and neck of the bladder, with a diameter of about 4 × 3 cm^2^. After electrical resection to the normal prostate tissue, the surrounding and deep parts of the prostate tissue were also resected. There was transient fluctuation of intraoperative blood pressure, up to 170/110 mm Hg. The prostate cancer was successfully resected. Ectopic prostate pheochromocytoma was identified by histological examination (Fig. 5). On immunohistochemical examination, the tumor cells were positive for CgA, SyN, and CD56.

In the postoperative period, all of his symptoms completely and immediately disappeared. Serum metanephrine and normetanephrine were within normal range. Over a 21-month follow-up period, a repeat abdominal CT scan did not show any evidence of recurrence.

## 3 Discussion

Pheochromocytoma is a chromaffin cell-derived neuroendocrine tumor usually present in the paraganglion cells or adrenal medulla, with an incidence of 0.1% to 0.3% in the hypertensive population.^[5]^ The common clinical symptoms of pheochromocytoma are paroxysmal hypertension, palpitations, tachycardia, headache, anxiety, and nervousness.^[6]^ It has also been reported to mimic insulin-dependent diabetes mellitus. ^[7]^ The varied signs and clinical presentations are important for clinicians suspecting pheochromocytoma. Increased catecholamine production is a marker of pheochromocytoma. This includes elevated serum metanephrine, serum normetanephrine, or 24-hour urine catecholamine. In our case, laboratory tests revealed elevations in all these parameters which strongly suggested pheochromocytoma. Furthermore, our patient complained of palpitations and anxiety on urination, which conformed to the symptoms of catecholamine excess. However, it is challenging to diagnose a prostrate pheochromocytoma by either CT or MRI.

The differential diagnosis of urinary and elevated serum catecholamines includes pheochromocytoma, neuroblastoma, gangliocytoma, congestive heart failure, and various stress states. Pheochromocytoma should be suspected when plasma or urinary normetanephrine and metanephrine are elevated and combined with typical clinical symptoms of pheochromocytoma. More than 95% of pheochromocytomas are found by either CT or MRI.^[8]^ Extra-adrenal pheochromocytomas are rare in adults, but account for 30% to 40% of pheochromocytomas in children.^[9]^ Pheochromocytoma of the urinary system accounts for less than 1% of extra-adrenal pheochromocytomas.^[10]^ As extra-adrenal pheochromocytomas are also developed from the chromaffin cells, most of these tumors are infradiaphragmatic within the para-aortic region, especially the abdomen and pelvis.^[11]^ Most reported urinary tract pheochromocytomas are located in the bladder.^[12]^ To our knowledge, ectopic prostate pheochromocytoma has rarely been reported.

Most pheochromocytomas are benign lesions, and only 2% to 13% of them are malignant. Some scholars have found that about 10% of ectopic pheochromocytomas are malignant.^[3]^ It was also reported that ectopic pheochromocytomas are more vascularly aggressive and locally invasive, and more likely to metastasize to lymph nodes.^[14]^ However, with the correct diagnosis and treatment, most patients have a good prognosis.

Surgical excision of the tumor is the definitive treatment for pheochromocytoma.^[13]^ Laparoscopic surgery is the first line therapy for resection of adrenal or extra-adrenal pheochromocytoma.^[14]^ Liu et al^[15]^ reported that a larger tumor diameter was associated with a higher risk of intraoperative bleeding and longer intensive care unit stays. In our case, the tumor size was small, so laparoscopic surgery was first recommended, but refused by the patient. Since we did not find metastatic tumor in our patient, we decided to perform transurethral resection of the prostate pheochromocytoma. To our knowledge, this operative technique for the treatment of ectopic prostate pheochromocytoma has not been previously reported.

It has been reported that pheochromocytoma surgery is associated with high rates of morbidity and mortality.^[16]^ As pheochromocytomas may secrete a great deal of circulating catecholamines, adequate preoperative medical treatments are recommended for all patients to minimize operative and postoperative complications due to hypertensive crises. In our opinion, there are advantages and limitations in using TURP vs laparoscopy in surgically excising ectopic prostate pheochromocytoma. TURP has lower technical requirements than laparoscopy and can be carried out in the basic level hospital with electric cutting equipment. It introduces less trauma, and the complications of urinary incontinence, retrograde ejaculation, and erectile dysfunction after operation may be greatly reduced. However, compared with laparoscopic radical prostatectomy, there is greater stimulation of the tumor and a longer surgical time, which increases the difficulty of anesthesia. In the present case, the patient was preoperatively normotensive, but we still administered 10 mg of phenoxybenzamine before surgery to avoid intraoperative blood pressure fluctuations.

Finally, the present case serves to highlight several learning points worthy of notice. Firstly, urologists should be aware that the prostate is a rare but possible site of ectopic pheochromocytoma, so pelvic imaging such as CT or MRI should be included as part of the routine protocol for the screening of pheochromocytoma. Secondly, laparoscopic surgery is the first line therapy for management of pheochromocytomas, but TURP may be a viable alternative for benign pheochromocytoma.

## Author contributions

**Data curation:** Wen-feng Wu, wenbin zhang, wh lei.

**Investigation:** JIN ZHOU, wenbin zhang.

**Resources:** JIN ZHOU.

**Writing – original draft:** wh lei.

**Writing – review & editing:** JUN Xin.
